# Synergistic activation of genes promoting invasiveness by dual deprivation in oxygen and nutrients

**DOI:** 10.1111/iep.12464

**Published:** 2023-01-24

**Authors:** Charly Jehanno, Yann Le Page, Gilles Flouriot, Pascale Le Goff, Denis Michel

**Affiliations:** ^1^ University of Rennes Inserm, EHESP, Irset UMR 1085 Rennes France; ^2^ Department of Biomedicine University of Basel Basel Switzerland

**Keywords:** breast cancer, epithelial–mesenchymal transition (EMT), gene expression, hypoxia, metastasis

## Abstract

By depriving cancer cells of blood supplies of oxygen and nutrients, anti‐angiogenic therapy is aimed at simultaneously asphyxiating and starving the cells. But in spite of its apparent logic, this strategy is generally counterproductive over the long term as the treatment seems to elicit malignancy. Since a defect of blood supply is expected to deprive tumours simultaneously of oxygen and nutrients naturally, we examine here these two deprivations, alone or in combination, on the phenotype and signalling pathways of moderately aggressive MCF7 cancer cells. Each deprivation induces some aspects of the aggressive and migratory phenotypes through activating several pathways, including HIF1‐alpha as expected, but also SRF/MRTFA and TCF4/beta‐catenin. Strikingly, the dual deprivation has strong cooperative effects on the upregulation of genes increasing the metastatic potential, such as four and a half LIM domains 2 (FHL2) and HIF1A‐AS2 lncRNA, which have response elements for both pathways. Using anti‐angiogenic agents as monotherapy is therefore questionable as it may give falsely promising short‐term tumour regression, but could ultimately exacerbate aggressive phenotypes.

## 1 INTRODUCTION

When deprived of nutrient and oxygen resources, beyond a critical size, tumours stimulate their own vascularization.^1^ Therefore, naturally anti‐angiogenic treatments are expected to forbid the growth of tumours, by preventing neovascularization and the supply of oxygen and nutrients. Despite this rationale, the approach often gives disappointing results because of an increase in recurrences.^2^, ^3^, ^4^, ^5^ To understand this drawback, we tested the effects of deprivations in (1) oxygen, (2) nutrients and (3) both. Indeed, to be relevant, experiments mimicking anti‐angiogenic conditions must carry out this dual deprivation. We found that the withdrawal of each ingredient favours dedifferentiation and acquisition of a migratory phenotype by activating interfering mechanisms involving HIF1‐alpha (HIF1A), the central actor of the response to hypoxia, SRF/MRTFA, known to reprogramme cells and to mediate mechanical genetic transduction leading to contractile activities and TCF4/beta‐catenin (CTNNB1), also clearly involved in epidermal–mesenchymal transition (EMT). Strikingly, when applied simultaneously, the lack of oxygen and nutrients induces‐in a strongly cooperative manner‐the expression of genes promoting invasiveness and increasing the metastatic potential, such as the migration gene FHL2 and the mesenchymal marker HIF1A‐AS2. Finally, these pathways influence the activity of each other and potentiate the activity of HIF1A. Together, these results clearly suggest that the anti‐angiogenic strategy could dramatically favour cancer cell dissemination.

## 2 MATERIALS AND METHODS

### 2.1 Cell culture and transfection

The human breast cancer MCF7 cells or MCF7 cells stably expressing MKL1‐CA^6^ were grown in Dulbecco's modified Eagle medium (DMEM‐GIBCO) containing 10% foetal bovine serum (FCS‐Biowest) and antibiotics (GIBCO) at 37°C under 5% CO_2_. Before the experiments, the cells were maintained for 24 h in DMEM with 2% dextran/charcoal‐stripped FCS (dcFCS‐Biowest) and then further incubated for 24 h in the specified medium. The poor medium (PM) is DMEM without pyruvate, glutamine and glucose but with 2% dcFCS. Transfection experiments of plasmid DNA or si‐RNA were performed as previously described.^6^ The wound healing assay was performed using an Ibidi® chamber according to the manufacturer's instructions. After verifying that identical results on cell dissociation and induction of EMT characteristics were obtained with gaseous hypoxia and by the addition of 200 μM CoCl_2_, we used this latter chemical treatment in the following experiments presented herein to facilitate cotreatments and long‐term treatments. Immunofluorescence experiments were performed as previously described.^6^

### 2.2 Plasmid, siRNA and antibodies

The p3Xflag‐MKL1‐N200 expression vector was kindly provided by Professor R. Prywes (Colombia University, New York, NY, USA). It encodes a constitutively nuclear and active form of MRTFA (MRTFA‐CA). pcDNA3‐S33Y CTNNB1 (#19286), M50 Super 8x TOPFlash (TCF4‐Luc #12456) and HRE‐luciferase (#26731) were purchased from Addgene. Human MRTFA (ON‐TARGETplus® SMARTpool®) and esiRNA human HIF1A were purchased from Thermo Scientific and Sigma respectively. Antibodies against the following proteins were used: CTNNB1 (ab6302 Abcam), CDH1 (ab15148 Abcam), ERK (extracellular signal‐regulated kinase 1); K‐23 ESR1 (HC‐20, SC‐543, Santa Cruz Biotechnology), FHL2 (ab12327; Abcam), HIF1A (610958 BD Biosciences), MRTFA (ab113264 Abcam) and phalloidin‐iFluor 594 (lab176757; Abcam).

### 2.3 Western blotting

Cells were lysed by sonication in a denaturing buffer (4% SDS, 200 mM dithiothreitol, 20% glycerol, 0.001% bromophenol bleu and 100 mM Tris–HCl pH 6.8). Protein extracts were separated by SDS–PAGE, transferred to nitrocellulose membrane and probed with specific primary antibodies. After incubation with horseradish peroxidase‐conjugated secondary antibody, complexes were detected with chemiluminescent HRP substrate (Immobilon Western, Millipore).

### 2.4 RNA extraction and RT‐qPCR

Total RNA was extracted from cells using the RNeasy kit (Qiagen) according to the manufacturer protocol. Five hundred nanogram of RNA were reverse transcribed using random primers and the iScript Reverse Transcription Supermix was purchased from Biorad. Quantitative real‐time PCRs were performed with CFX384 touchTM real‐time PCR detection system using the iQTM SYBR Green supermix from Biorad. The following primer sequences were used: ACTG2: forward GCGGAAGTACTCAGTCTGG, reverse CACTTCCTGTGGACAATGGA; FHL2: forward GGCAAGAAGTACATCCTGCG reverse CCACCAGTGAGTTTCTGCAC, HIF1A‐AS2: forward AAAGCTTGGGCAAATTATTCA, reverse TGAATGGGATGAGTGAAGCA; SRF: forward CATCCCTTGGGCCATCTGT, reverse GCAGTTCAGCTCCACCAGAT; SNAI2: forward CAAGGACACATYAGAACTCACAC, reverse AGGTTTTGGAGCAGTTTTTGCAC; and VEGFA: forward AGGAGGAGGGCAGAATCATCA, reverse CTCGATTGGATGGCAGTAGCT.

### 2.5 ChIP‐seq data

The genomic binding sites for SRF in MCF7 cells were from ENCODE DCC (Stanford, GEO access: GSM1010839). The identification of MRTFA‐binding sites was performed using ChIP‐seq experiments described in.^7^ The identification of TCF4‐binding regions in the human genome was performed using MCF7 cells (Peggy Farnham lab, USC. GEO:GSM945859). The HIF1A‐binding sites were determined by ChIP‐seq in MCF7 cells (David R. Mole lab. University of Oxford GEO access: GSM700944). ChIP‐Seq data on the presence of CTNNB1 are available in SW480 cells GSE5392735. For functional annotation, we collected bed files provided in the publication corresponding to genomic regions specifically bound by the corresponding transcription factors or cofactors. Using the online GREAT platform (http://great.stanford.edu/) and using the following settings (5000 bp upstream and 1000 bp downstream, with up to 30 kb extension), the list of genes bound by selected transcription factors and cofactors was generated.

### 2.6 Quantification

Immunostaining signals were quantified from fluorescence microscopy images by averaging the intensity of more than a thousand cells using an automatic Fiji plugin. After nucleus identification using DAPI labelling and background subtraction, total fluorescence was extracted from the picture obtained with the fluorescent antibody. Superscripts on histograms correspond to Mann–Whitney *U* tests. RT‐PCR values are expressed as the mean fold induction compared to controls of four independent experiments; *p* < .05 with the Mann–Whitney *U* test.

## 3 RESULTS AND DISCUSSION

### 3.1 Effects of CoCl_2_ and poor medium on HIF1A and cellular phenotypes

**FIGURE 1:**
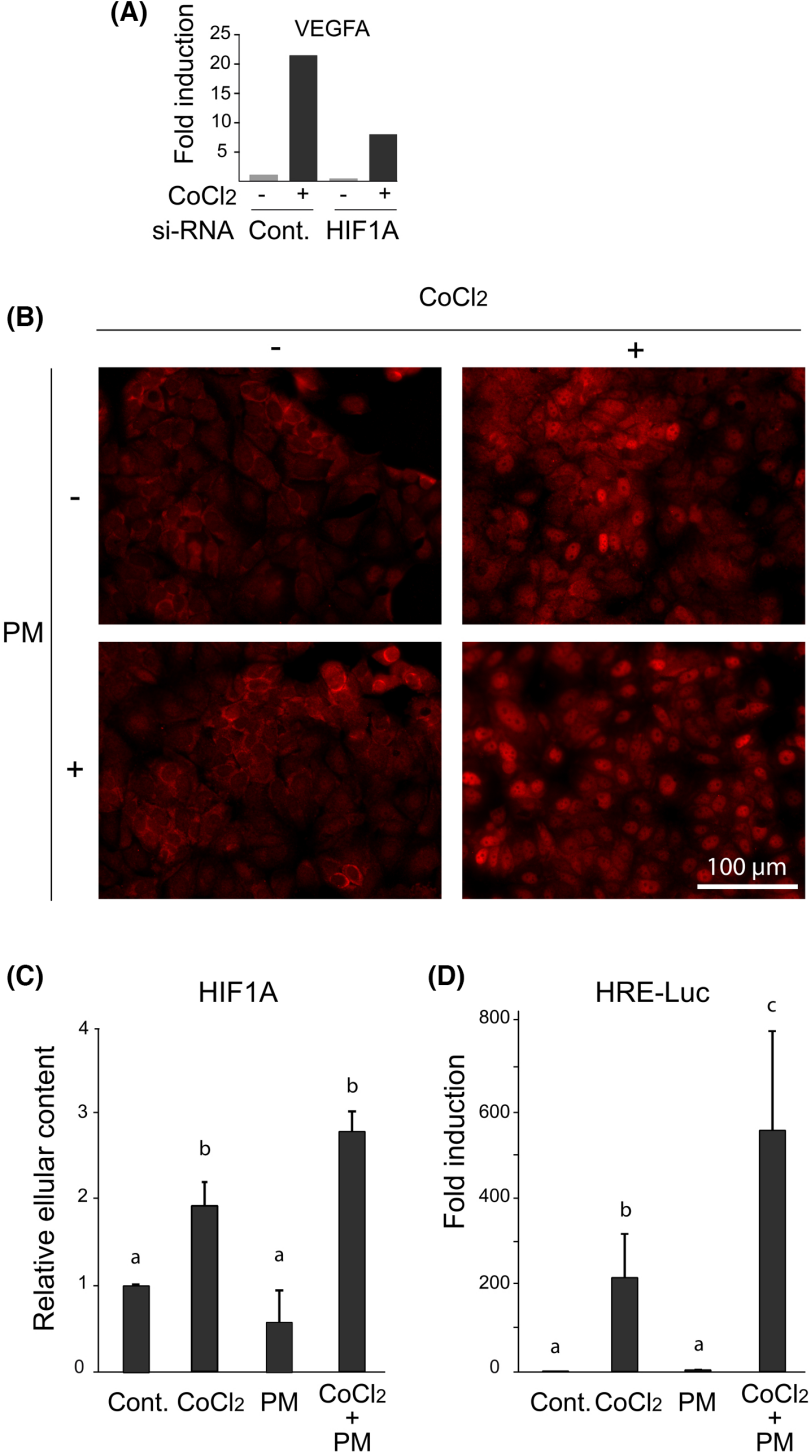
Effects of CoCl_2_ and nutrient‐poor medium (PM) alone or in combination, on the subcellular location, the cellular content and the transcriptional activity of HIF1A. (A) si‐RNA experiment aimed at verifying that CoCl_2_ induces HIF1A‐mediated expression of the angiogenetic factor VEGFA mRNA measured by RT‐PCR. (B) Effects of CoCl_2_ and PM on the subcellular distribution of HIF1A. (C) Total cellular content of HIF1A following CoCl_2_ and/or PM treatments. (D) HIF1A‐responsive element (HRE)‐driven transcription in response to CoCl_2_ and/or PM treatments. Columns with different superscripts differ significantly (*p* < .05).

The molecular pathways involved in the response to hypoxia have long been identified as regular components of EMT, regardless of the primary mode of induction of this multifaceted cellular activity, which explains why hypoxia can itself enhance EMT. Hypoxia is one of the expected consequences of a lack of blood supply, which is itself logically concomitant with nutrient deprivation. These two deprivations are therefore associated in the case of anti‐angiogenic treatment, so it is crucial to test their joint action. To examine the impact on EMT of nutrient deficiency, alone or in conjunction with hypoxia, we selected the MCF7 mammary human cell line because it is not aggressive and retains marked characteristics of epithelial differentiation, whose loss can be easily detected. Especially when grown to confluency, these cells form pseudo‐epithelial layers with strong intercellular contacts. We tested the possible interferences of poor medium (written in abbreviated form PM and concretely defined herein as a low‐serum, glucose‐free, glutamine‐free and pyruvate‐free medium) with the major regulator of hypoxia, namely HIF1A. As chemical treatments with CoCl_2_, known to inhibit prolyl hydroxylases and stabilize HIF‐1,^8^ would be technically easier than gaseous hypoxia in our model, we first verified that they mimic hypoxia in these cells through the upregulation of its most representative marker, VEGFA, and that HIF1A is involved in this regulation, as controlled by siRNA. In the RT experiment of Figure 1A quantifying mRNA contents, in the presence of normal HIF1A (control siRNA unrelated to HIF1A), CoCl_2_ leads to a 20‐fold increase in VEGFA, whereas the anti‐HIF1A si‐RNA reduces this induction to 5‐fold, showing that HIF1A mediates, at least in part, the effect of CoCl_2_ on the most emblematic marker for hypoxia VEGFA. Figure 1 shows the different functional facets of HIF1A activation influenced by PM, including its nuclear enrichment, an increase in its cellular content (Figure 1B) and its transcriptional activity (Figure 1C). The increase in its cellular content may result from a stabilization according to its classical mode of activation. The nuclear translocation of HIF1A is clearly associated with its transcriptional activity, as determined by transient expression of an HRE (HIF‐responsive element)‐Luc reporter plasmid. PM stimulates the effects of CoCl_2_ on the nuclear translocation and transcriptional activity of HIF1A but has little impact on its own in the absence of CoCl_2_ (Figure 1D).

### 3.2 Candidate molecular actors mediating the effects of hypoxia and PM

**FIGURE 2:**
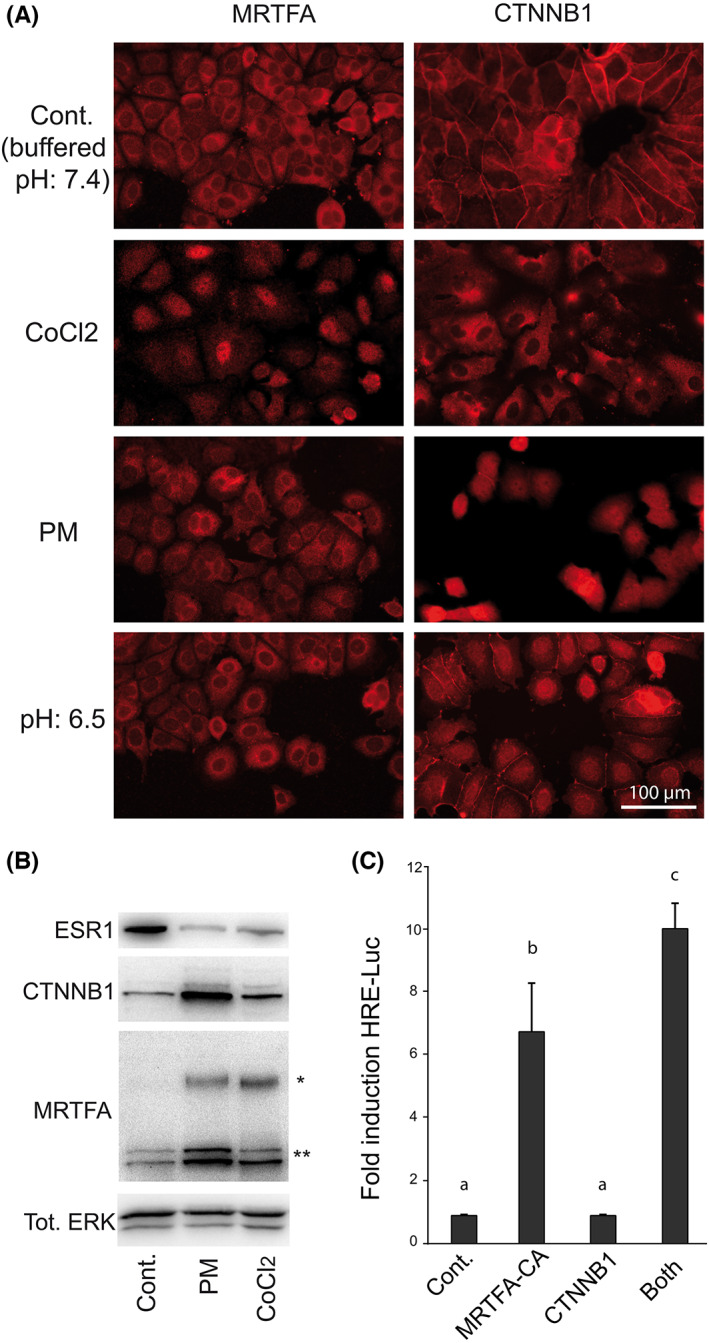
MRTFA and CTNNB1, candidate molecular actors for mediating the effects of CoCl_2_ and/or PM. (A) Differential effects of CoCl_2_ and/or PM and acidic medium on the nuclear translocation of MRTFA and CTNNB1. Acidosis (pH:6.5) is tested because it is an expected consequence of microenvironmental modifications by mesenchymal cells. (B) Immunoblotting detection of MRTFA and CTNNB1 in cells submitted to CoCl_2_ and PM. Note that the long (*) and short (**) MRTFA isoforms are differentially regulated. The three tracks shown on each panel are contiguous in the original transfer membranes. (C) HRE‐mediated transcription in cells cotransfected with either MRTFA‐CA or CTNNB1. Columns with different superscripts differ significantly (*p* < .05).

A previous study^6^ and preliminary experiments led us to select the SRF/MRTFA and TCF4/CTNNB1 signalling pathways as possible relays of the effects described above. In the literature, certain phenotypic changes induced by hypoxia are mediated by the SRF/MRTFA‐dependent contractile programme, clearly associated with EMT,^9^ either directly through RhoA‐ROCK1 signalling already known to be activated by hypoxia,^10^ or following actin polymerization.^11^ In addition, MRTFA has recently been shown to erase the initial cellular differentiation status^12^ and to favour the stemness of breast cancer cells.^13^ Another central actor of EMT^14^, ^15^ and invasion^16^ is CTNNB1. It has been shown to enhance HIF1A‐mediated transcription,^17^ but has also been proposed to mediate the effect of 2‐deoxyglucose,^18^ a competitive inhibitor of glucose uptake by cells, which is expected to mimic to some extent the PM. Moreover, a well‐established consequence of tissular disruption during EMT is the release of CTNNB1.^19^ Finally, the nuclear location of CTNNB1 has previously been shown to be associated with the acquisition of invasiveness in the MCF7 cells studied here.^20^ The experimental tests and ChIP‐seq data described below strongly support the validity of these candidates. A simple study of the cellular distribution of MRTFA and CTNNB1 was first conducted by immunocytochemistry, which shows interesting behaviours and different effects of CoCl_2_ and PM. The nuclear translocation of MRTFA is stimulated by CoCl_2_ while that of CTNNB1 is triggered by PM. As shown in Figure 2A, CTNNB1 staining, which is mainly located at the intercellular junctions in control conditions, becomes cytoplasmic in cells treated with CoCl_2_ and mainly nuclear in cells cultured in PM.

**FIGURE 3:**
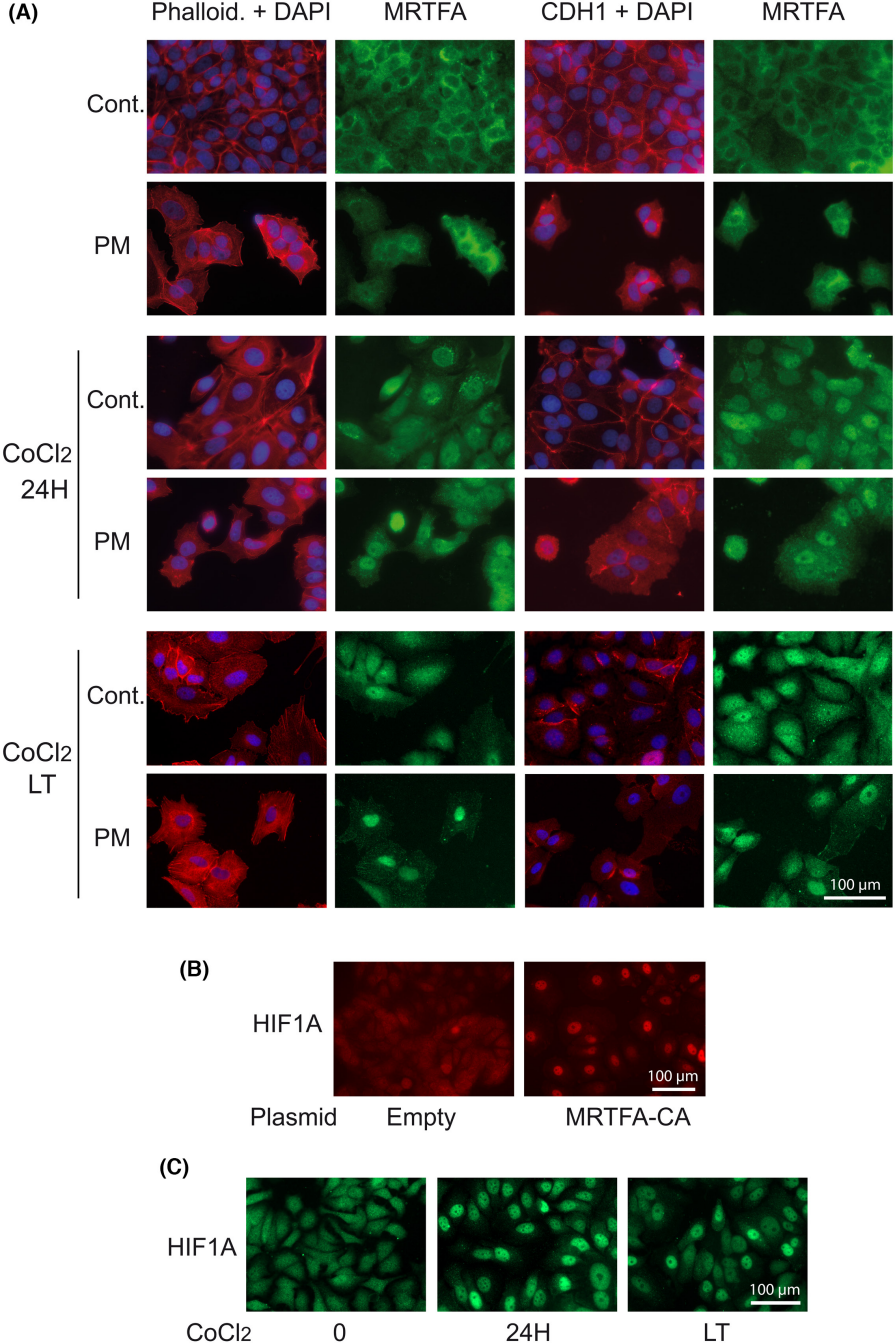
(A) Effects of short (24 h) or long (LT: >1 month) treatments with CoCl_2_, alone or in combination with PM, on the polymerization of filamentous Actin (phalloidin staining) and the redistribution of E cadherin (CDH1). The nuclear enrichment of endogenous MRTFA is shown in parallel. Twenty‐four‐hour CoCl_2_ and PM treatments both lead to cell–cell disjunction. A more pronounced nuclear translocation of MRTFA is obtained with CoCl_2_ compared to PM. (B) Stable expression of constitutively active MRTFA leads to the permanent nuclear localization of HIF1A. (C) Effect of short‐ and long‐term treatments with CoCl_2_ on HIF1A nuclear accumulation.

These experiments, however, do not fully recapitulate the expected effects of a defect in blood supply and irrigation of tumours because they were carried out in a buffered culture medium. To be complete, it would be necessary to examine the effects of acidosis. Indeed, one of the stereotyped facets of EMT is the metabolic reprogramming called the Warburg effect, from respiratory (oxidative phosphorylation) to fermentative (glycolysis) whose main by‐product, lactate, is exported in the extracellular space, leading to the acidification of the extracellular medium in the vicinity. In the literature, low pH has been shown to favour the migratory potential of cancer cells in two ways: in the cell, low pH stimulates the action of key enzymes,^21^, ^22^ while after proton export in the medium, low pH contributes to the dismantlement of the extracellular matrix,^23^ which can explain how acidosis can promote tumour invasion.^24^ We have tested the effect of acidosis specifically in the absence of hypoxia by replacing the standard buffered medium with an acidic medium (pH: 6.5). Although CoCl_2_ in buffered medium induces the nuclear translocation of MRTFA, no such translocation is obtained with low pH alone. By contrast, CTNNB1 previously located at the cellular contours becomes strongly enriched in the nucleus (Figure 2A). This observation corroborates the model described by Chen et al.^25^ that the disruption of cell–cell contacts could lead to the intracellular release of CTNNB1 previously trapped in adherens junctions. In addition to their nuclear translocation, we then measured the abundance of MRTFA and CTNNB1 by immunoblotting (Figure 2B). The intracellular content of CTNNB1 strongly increases in cells cultured in PM but less than with CoCl_2_. According to the literature, an increase in CTNNB1 concentration may result from a decreased degradation by the ubiquitin proteasome after the inactivation of certain of its ubiquitin ligase(s). Contrary to CTNNB1, the long form of MRTFA is induced more by CoCl_2_ than by the PM (Figure 2B). The effects of the treatments on the nuclear translocation and the abundance of CTNNB1 and MRTFA are parallel (compare Figure 2A and Figure 2B). A subtlety, however, appears in our immunoblotting experiment since the anti‐MRTFA antibody reveals several forms of this protein. Shorter ones, possibly derived from an internal transcription initiation,^26^ are increased in PM, in a manner similar to that of CTNNB1. This result will prove remarkably consistent with the genomic recruitment of MRTFA and CTNNB1 (see below). Since the loss of epithelial phenotype of MCF7 cells is also characterized by the disappearance of the oestrogen receptor alpha (ESR1), we verified that this phenomenon is also obtained with CoCl_2_ and PM (Figure 2A). Finally, in the absence of treatment, simple cotransfections with MRTFA‐CA or CTNNB1 induce HIF1A‐mediated transcription such as CoCl_2_ or PM respectively (compare Figure 1D and Figure 2C). In these experiments aimed at distinguishing their different signalling pathways, the treatments with CoCl_2_ and PM were applied separately, but to test a possible synergistic effect between these pathways, combined treatments were then tested. The phenotypic modifications observed by microscopy (Figure 3), including intercellular dysjunction and acquisition of a more stellate and less epithelial morphology, evoke epithelial–mesenchymal transition (EMT).

**FIGURE 4:**
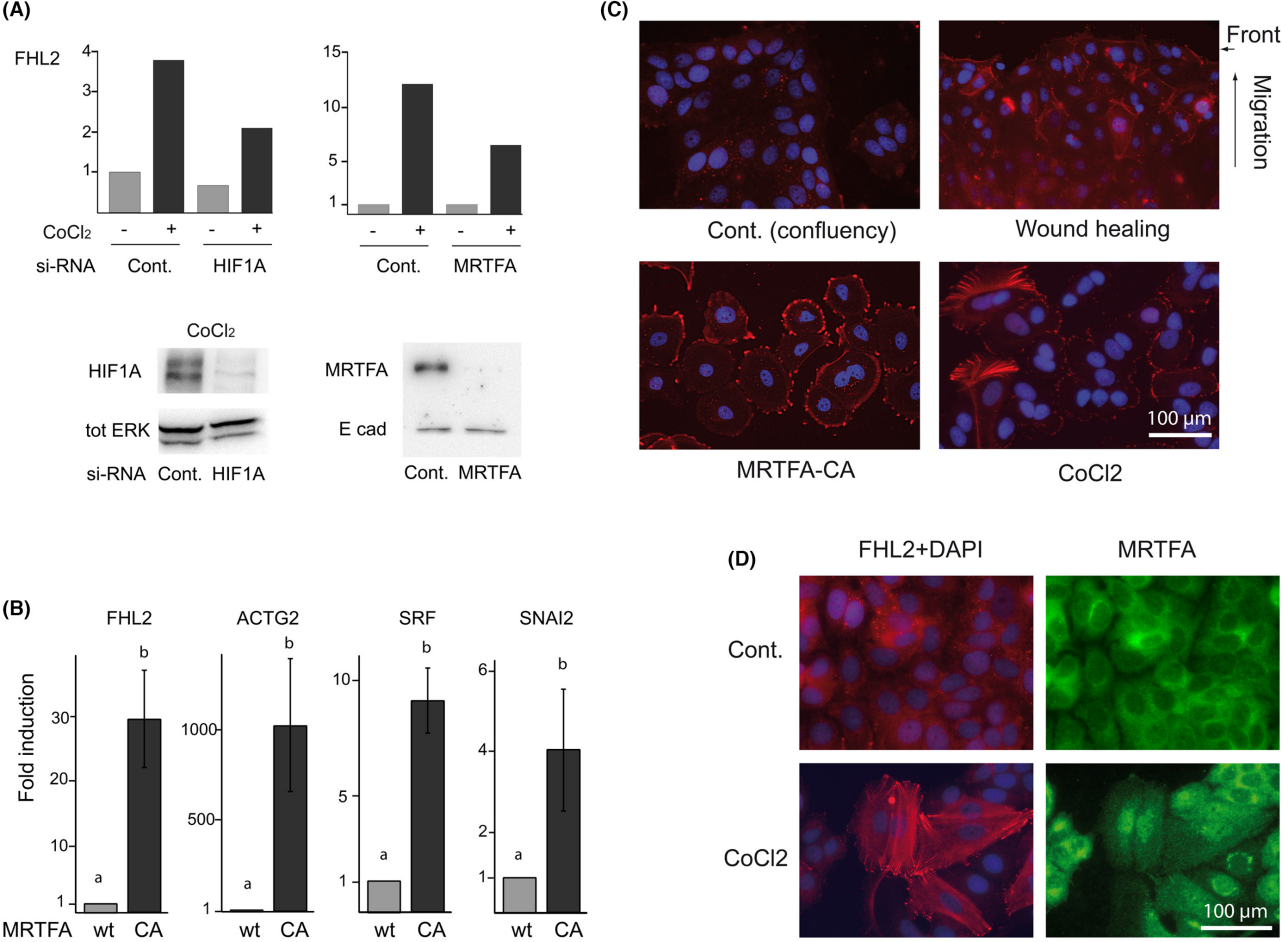
Regulation of FHL2. (A) si‐RNAs directed against HIF1A and MRTFA inhibit the expression of FHL2 by CoCl_2_. The bottom panels are immunoblots showing the capacity of the si‐RNAs to prevent the induction by CoCl_2_ of HIF1A and MRTFA. (B) Upregulation of FHL2, ACTG2, SRF and SNAI2 mRNAs in MCF7 cells stably expressing MRTFA‐CA. Columns with different superscripts differ significantly (*p* < .05). (C) Immunodetection of FHL2 in various conditions showing its accumulation in the leading migratory cells in wound assays (top right panel) and its accumulation in the nuclei and the borders of cells either overexpressing MRTFA‐CA or treated with CoCl_2_ (bottom panels). (D) Immunodetection of FHL2 (red) showing its overexpression and accumulation into bundles and focal adhesion points, precisely in the cells with a nuclear enrichment of MRTFA (green).

Both CoCl_2_ and PM treatments trigger the well‐established primary mark of EMT: dismantling of the intercellular contact marker CDH1 (E‐cadherin). CDH1 is present in epithelial cell‐to‐cell junctions close to actin filaments, and its disruption can be responsible for the release of CTNNB1 from cellular junctions observed in Figure 3A. Actin is redistributed accordingly, switching from cell borders to intracellular, sometimes in the form of stress fibres in the most transformed cells. We measured the polymerization of actin which is a well‐established mode of activation of MRTFA,^11^ and accordingly, modifications of the subcellular location of MRTFA are observed. The nuclear translocation of MRTFA clearly follows the intensity of phalloidin staining (Figure 3A). MRTFA, which is mainly cytoplasmic in control cells, is strongly relocated in cells treated with CoCl_2_, but less markedly enriched in the nuclei of cells cultured in PM, despite their stronger cell–cell dissociation. However, in some cells intensely stained with fluorescent phalloidin, a nuclear translocation of MRTFA is noticeable. With CoCl_2_, the nuclear enrichment of MRTFA is more obvious even when the cells begin to dissociate (Figure 2A). Hence, PM stimulates CoCl_2_‐induced nuclear translocation of both MRTFA (Figure 3A) and HIF1A (Figure 1B). The long‐term treatment with CoCl_2_ induces a stabler nuclear location of MRTFA while causing a less pronounced nuclear translocation of HIF1A (Figure 3C). The weaker nuclear relocation of MRTFA in cells cultured in PM, which contrasts with the severe phenotypic impact of this treatment, suggests the involvement of other signalling mechanisms, of which one could be CTNNB1 as supported by the results of Figure 2. PM induces cellular dissociation even more rapidly and markedly than CoCl_2_ but is not as potent for inducing the nuclear translocation of MRTFA. Although less effective in inducing acute genetic responses, fluorescence microscopy (Figure 3A, bottom panels) suggests that a long‐term treatment with CoCl_2_ stabilizes the nuclear location of MRTFA. We, therefore, selected this condition for the following experiments. Very interestingly, the functional relationships between the hypoxia pathways and MRTFA seem to be reciprocal. The activation of HIF1A by CoCl_2_ (Figure 1A) is accompanied by a nuclear relocation of MRTFA, while conversely, the forced expression of a constitutively active mutant version of MRTFA induces the nuclear localization of HIF1A (Figure 3B). This cross‐talk, to which others will be added later, could create a positive feedback stabilizing the cellular response. The interrelation between HIF1A and MRTFA is clearly illustrated by the behaviour of FHL2, a typical target gene of MRTFA. Anti‐MRTFA as well as anti‐HIF1A siRNAs significantly decrease the induction of FHL2 by CoCl_2_ (Figure 4A), showing that MRTFA actually mediates, at least in part, the effects of hypoxia.

Accordingly, the forced expression of the MRTFA‐CA mutant clearly upregulates FHL2 and other SRF/MRTFA target genes as ACTG2 and SRF itself (Figure 4B). FHL2 accumulates precisely in the cells enriched in nuclear MRTFA (Figure 4D), consistent with the regulation of this gene by the SRF/MRTFA pathway. The roles of FHL2 in contractile and migratory phenotypes are reflected by its subcellular organization and its accumulation at the peripheral adhesion points. Indeed, this multifunctional adaptor protein has been shown to simultaneously inhibit the cell cycle and stimulate tumoural invasion: it is (1) a cog in the mechanical phenomena of migration, working in the focal adhesion points with partners such as filamin; and (2) a transcriptional regulator participating to cell cycle arrest, in part by promoting the expression of CDKN1A (P21). In keeping with the previous results, MCF7 cells overexpressing MRTFA‐CA upregulate FHL2 (Figure 4C, bottom left panel) and systematically display a strong FHL2 staining similar to that obtained with CoCl_2_ (compare the bottom panels of Figure 4C). The link between migration and FHL2 is further illustrated by the selective presence of FHL2 in the front of migration in wound healing assays (Figure 4C, top right panel), in accordance with its roles in cell migration and wound healing.^27^ In this respect, the involvement of FHL2 in cancer is not a surprise considering that this pathology has been pertinently compared to a sort of overhealing,^28^ a notion confirmed by their largely overlapping genetic programmes.^29^

### 3.3 Joint transcriptional effects of CoCl_2_ and PM

**FIGURE 5:**
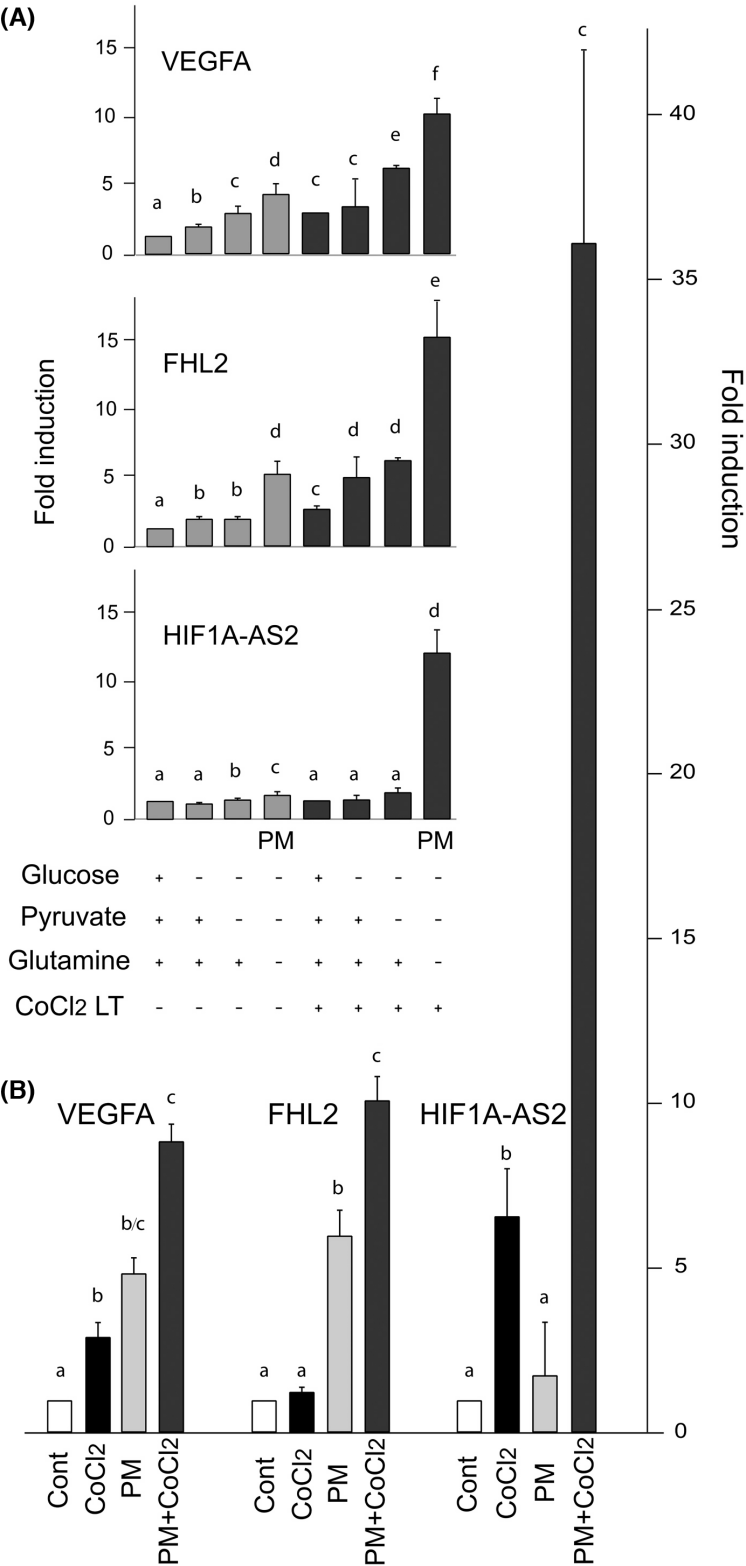
Effect of short‐ and long‐term treatments with CoCl_2_ and PM on VEGFA, FHL2 and HIF1A‐AS2 RNA accumulation. (A) Depletions of glucose, pyruvate and glutamine, in conjunction or not with long‐term treatment with CoCl_2_. (B) Short‐term treatment with CoCl_2_ with or without PM. Columns with different superscripts differ significantly (*p* < .05).

We quantified by RT‐PCR the effects of CoCl_2_, PM and both, on the cellular accumulation of the mRNAs encoding VEGFA, the traditional HIF1A target gene, FHL2, the established target of MRTFA and the noncoding RNA HIF1A‐AS2 involved in the establishment of a malignant phenotype.^30^, ^31^, ^32^ We tested short‐term (24 h) and long‐term (1 month) CoCl_2_ treatments because of their different effects on HIF1A and MRTFA nuclear enrichments (Figure 3), together with partial or complete nutrient deprivation. The long treatment, associated with depletions in glucose, pyruvate and glutamine, slightly overadditively induces VEGFA, but strongly overadditively induces FHL2 and HIF1A‐AS2 (Figure 5A). This synergistic induction is particularly clear for HIF1A‐AS2 and spectacular with a short treatment with CoCl_2_ (Figure 5B).

### 3.4 Comparison with genomic data

**FIGURE 6:**
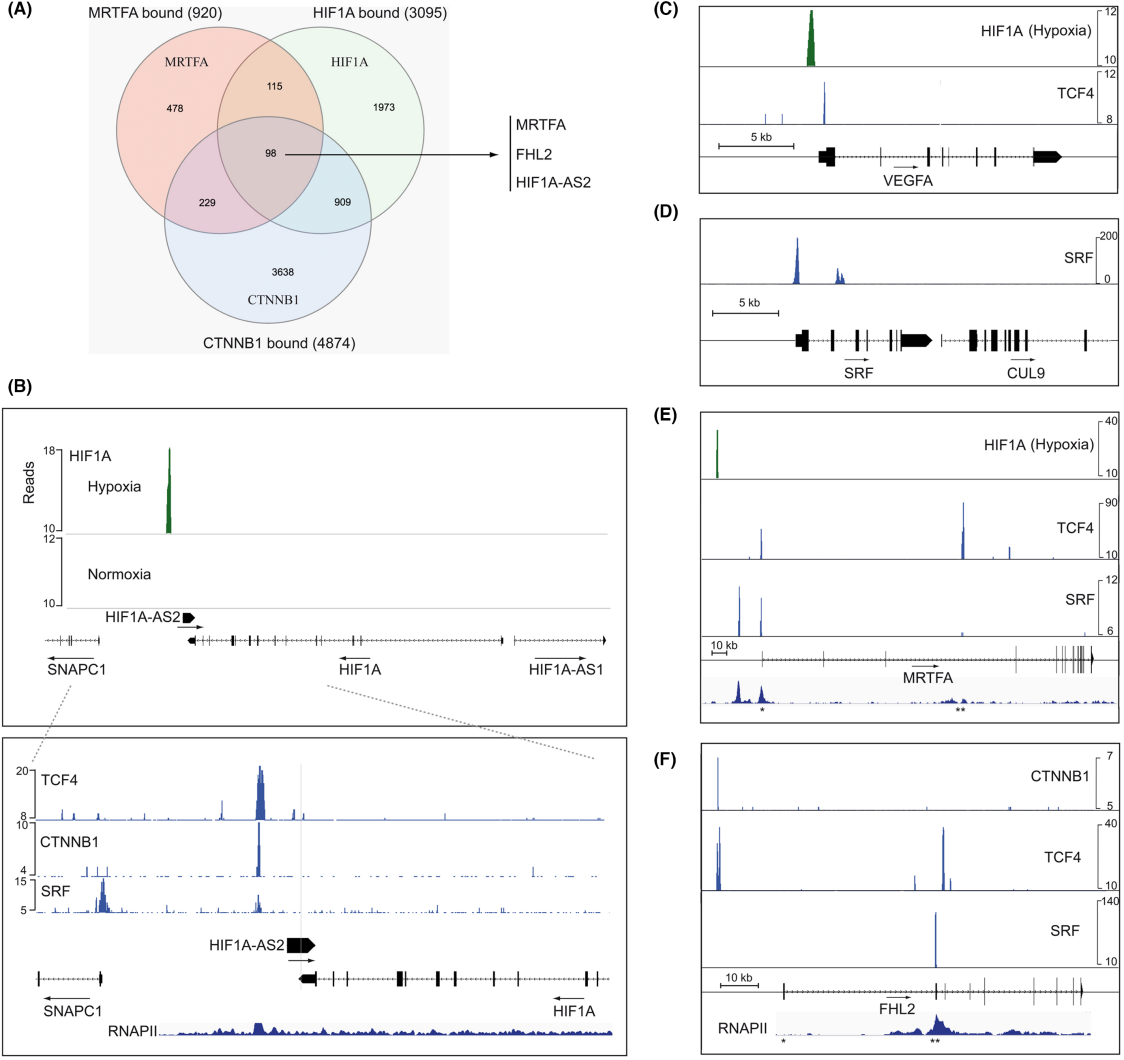
ChIP‐seqs. (A) Venn diagram showing the recruitment of MRTFA (MKL1), CTNNB1 (beta‐catenin) and HIF1A (HIF1alpha). A subset of 98 genes can be bound by these three factors. (B) Coexistence of recruitment sites for TCF4, CTNNB1, SRF and HIF1A in the proximal regulatory region of the HSF1A‐AS2 gene. Genomic recruitment sites in the proximal regulatory regions of (C) VEGFA (D) SRF, (E) MRTFA and FHL2 (F) genes. Accumulation of RNAPII reveals the presence of internal promoters (**) in FHL2 and MRTFA genes.

To evaluate the panel of genes that can be subjected to cross‐regulation by HIF1A, MRTFA and CTNNB1, we launched a meta‐analysis aimed at identifying genes with binding sites for these factors. The mere presence of a transcription factor‐binding motif, even near‐consensual, is insufficient to prove the actual fixation of this factor because it can be prevented by chromatin organization. Hence, we assessed the existence of functional binding sites extracted from ChIP‐seq data performed against these three factors. Using the GREAT online platform, we identified from bed files a shortlist of 98 genes whose regulatory elements are bound by HIF1A, MRTFA and CTNNB1, as shown in the Venn diagram of Figure 6A.

Strikingly, the three genes FHL2, MRTFA and HIF1A‐AS2 belong to this subset of genes. VEGFA was only found bound by HIF1A and CTNNB1 but not by MKL1. Functional annotation of genes sharing these corecruitments using the WikiPathway resource reveals that the top five cellular activities with combined scores higher than 15, including focal adhesion, integrin‐mediated cell adhesion, TGF‐beta signalling pathway and EGR/EGFR signalling pathway, are all associated with aggressiveness. We then focused our attention on the proximal regions of the few genes identified above to clearly visualize the recruitment peaks. In addition, we looked at the fixation relays of the MRTFA and CTNNB1 factors. Indeed, MRTFA and CTNNB1 are coactivators devoid of DNA‐binding domains but endowed with strong transactivation domains. CTNNB1 coactivates DNA‐bound TCF4 while MRTFA coactivates DNA‐bound SRF, whereas TCF4 and SRF are by themselves poorly transcriptionally active. An additional complication is that different coactivators are possible for SRF. It can, for instance, be activated by pro‐mitotic ELK and pro‐migratory MRTFA factors, the choice between these optional coactivators seems largely dictated by gene‐specific contexts,^33^ and the genes strongly upregulated in the present case have been shown to be preferentially regulated by MRTFA, as verified by the systematic screen of.^7^ The long noncoding HSF1A‐AS2 RNA can naturally not be visualized by immunocytochemistry, but the transcription factor recruitments near its transcriptional initiation site (Figure 6B) suggest that it has a central role in the system studied here. The HSF1A‐AS2 gene is located in close vicinity of HIF1A, encoding the master transcriptional regulator of hypoxia; but while HIF1A is itself poorly regulated at the transcriptional level, HSF1A‐AS2 is a transcriptional target for many signalling pathways including HIF1A itself (Figure 6B). This combination of DNA‐bound factors can explain the powerful synergy between these factors and the synergy between CoCl_2_ and PM treatments. Recent functional studies have established that HSF1A‐AS2 can control hypoxic pathways even in the absence of hypoxia^30^ and that the expression of this RNA promotes a marked metastatic mesenchymal phenotype.^31^ These properties may mediate the adverse effects of the dual oxygen and nutrient deprivation. In line with previous reports on the regulation of FHL2 by MRTFA,^34^ a proximal peak of recruitment of SRF is found in front of the second exon of the FHL2 gene (Figure 6F), centred on a consensual SRF‐binding site (5′‐ccttatatgg‐3′) and close to the main transcription start site, as verified by the accumulation of RNAPII in this region. When inserted in an enhancer‐less reporter plasmid, this element mediates more than 200‐fold induction by activated MRTFA (not shown). TCF4‐binding sites are also present in the vicinity, but more clearly in front of the first exon. This upstream TCF4‐binding site is validated by the superposed recruitment of CTNNB1 (Figure 6F). The coexistence of SRF/MRTFA and TCF4/CTNNB1‐binding sites could mediate the synergistic coregulation of FHL2 by these factors (Figure 5B). We did not find a clear HIF1A‐binding site near the FHL2 gene although it has been described as a HIF1A target gene.^35^ Instead, the upregulation of FHL2 by hypoxia could be indirect and mediated, for example, by the SRF/MRTFA pathway also involved in hypoxia. Interestingly, the MRTFA gene itself can be bound by SRF, TCF4 and HIF1A. The RNAPII ChIP‐seq also suggests the presence of two transcription initiation sites in the MRTFA gene. The conventional upstream promoter contains recruitment sites for HIF1A and SRF and a minor binding site for TCF4. In addition, an internal putative promoter between the exons 3 and 4, as in the study of^26^ and coinciding with accumulation of RNAPII, contains a major recruitment site for TCF4 (Figure 6E). These recruitments fairly agree with our measurements of MRTFA expression (Figure 2B), since CoCl_2_, which we have shown to induce MRTFA, mainly regulates the long form, in a self‐regulatory mechanism, while the PM rather induces the short isoform of MRTFA (Figure 2B). This short protein version has been otherwise involved in cellular transformation into myofibroblasts,^26^ a process requiring the massive synthesis of contractile machineries. The SRF/MRTFA transcription system appears remarkably self‐regulated since both SRF and MRTFA genes are targets of SRF (Figure 6D,E). The coexistence of TF recruitments on a promoter is not a sufficient condition for cooperativity which obeys complex and hardly predictable rules, but it explains at least in part the striking transcriptional results obtained for FHL2 and HIF1A‐AS2. A more modest cooperativity (factor of 1.3) is obtained for MRTFA.

## 4 CONCLUSIONS

**FIGURE 7:**
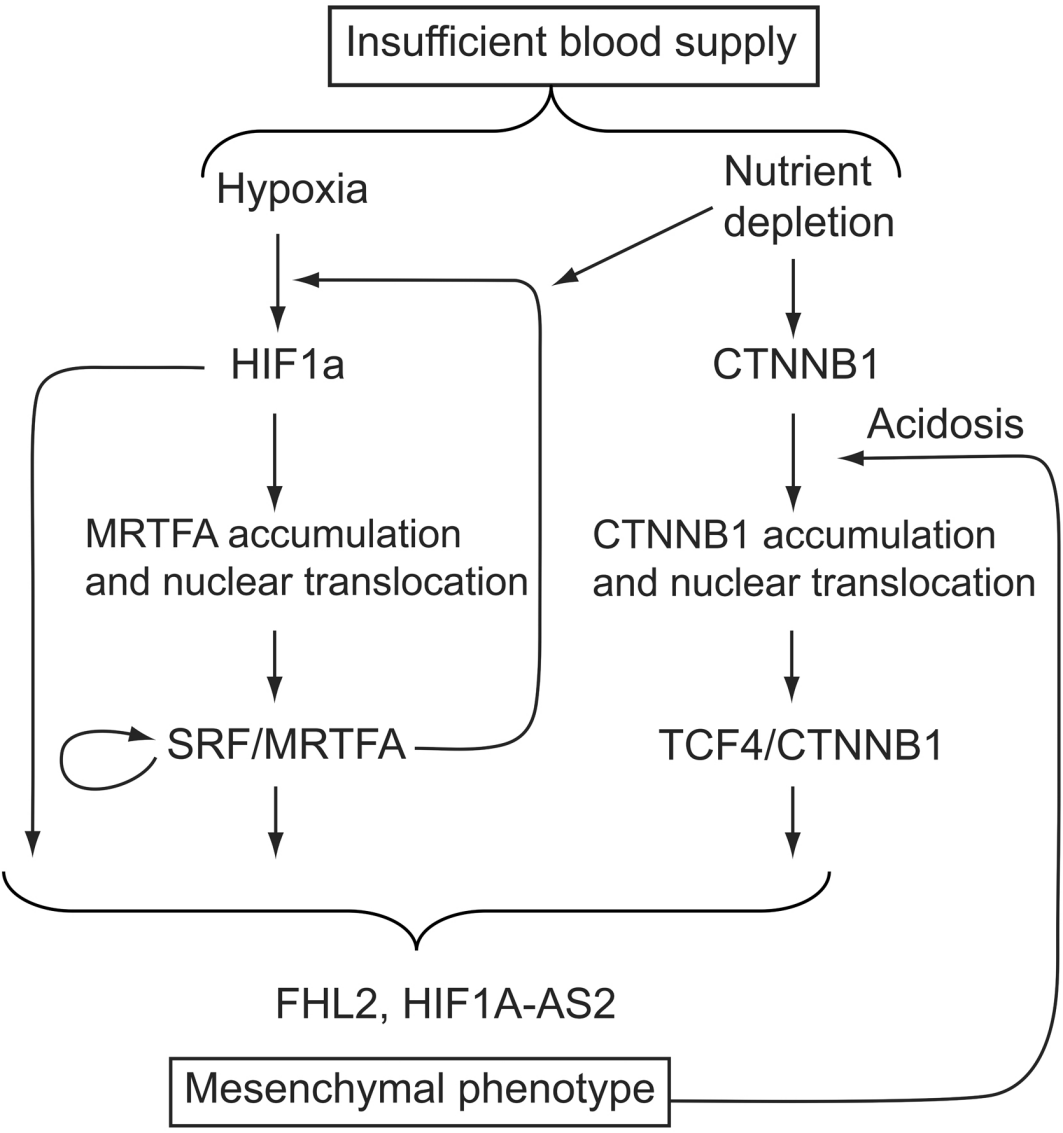
Schematized molecular consequences of the dual deprivation of oxygen and nutrients as a result of blood supply interruption.

Oxygen and nutrient deprivations are shown here to cooperate for promoting epithelial dedifferentiation and acquisition of a migratory phenotype of MCF7 cells, through activating at least three different pathways: HIF1A, SRF/MRTFA and TCF4/CTNNB1, as summarized in Figure 7.

These interfering pathways converge to key genes critical for the establishment of an aggressive phenotype, including FHL2 and HIF1A‐AS2. In addition to governing FHL2 and HIF1A‐AS2 expression, SRF/MRTFA and HIF1A are functionally connected. The convergence of pathways towards common target genes can explain (i) why they induce similar cellular behaviours and, more importantly, (ii) how they can act synergically in the induction of EMT in case of dual depletion of oxygen and nutrients, a situation precisely expected when cutting off blood supply. Moreover, once initiated, these responses are then modulated by various cross‐ and reciprocal regulations in the system, including positive feedbacks (Figure 7), and possibly negative feedbacks. For instance, FHL2, which is a target for MRTFA, has been shown upregulated by hypoxia/CoCl_2_ ^35^ but also reported to interact with HIF1A and repress its transcriptional activity.^35^ Besides, CTNNB1 enhances the transcriptional activity of HIF1A, but its own gene is repressed by HIF1A.^17^ In addition to regulate common target genes, the pathways defined here can reinforce each other. For instance, the CTNNB1 pathway was shown to be induced by acidosis, as confirmed here in Figure 2, which itself may be a consequence of the initiation of the Warburg effect by hypoxia, thereby self‐enforcing EMT in vivo. The present results could provide molecular bases underlying the questioning of the anti‐angiogenic therapy in the literature.^2^, ^3^ The pro‐tumoral effect of chronic hypoxia has long been reported: for example, in a study showing that long‐term intermittent hypoxia triggers the emergence of malignant cells starting from normal cells.^36^ Pathologists have long noticed that the cells embedded at the centre of solid tumours, which are the less well irrigated, display the most aggressive phenotype with stellate contours and are enriched in polymerized actin. Recent experiments cancelling blood supply strikingly confirmed the risk of hypoxia and nutrient deficiency caused by anti‐angiogenic approaches.^37^ The unintended consequences of starvation and hypoxia shown in the study of Wang et al.^37^ on migratory cellular behaviours appear in fact logical and adaptive and might have been anticipated. Indeed, faced with a lack of supply of oxygen and nutrients, the possible responses of cancer cells for their survival can be either (1) autophagy, enabling them to resist temporarily by drawing resources (carbon and energy) from their own constituents,^38^ or (2) mobility, leading them to adopt the nomadic and opportunistic consumer strategy of invaders. In this context, the anti‐angiogenesis strategy would be seriously counterproductive by inciting the few surviving cells to acquire a migratory phenotype and spread in the organism. Anti‐angiogenic treatments can first cause encouraging tumour regression, but can then increase the risk of recurrence possibly mediated by a direct influence of hypoxia on glycolysis^39^ and metastasis.^4^, ^5^, ^10^ The identification of candidate mechanisms involved in all these observations could help develop new strategies to circumvent these pitfalls.

## CONFLICT OF INTEREST

The authors declare no competing interest.
